# Other-Focused Approach to Teaching. The Effect of Ethical Leadership and Quiet Ego on Work Engagement and the Mediating Role of Compassion Satisfaction

**DOI:** 10.3389/fpsyg.2021.692116

**Published:** 2021-06-24

**Authors:** Ilaria Buonomo, Maria Luisa Farnese, Maria Luisa Vecina, Paula Benevene

**Affiliations:** ^1^Department of Human Sciences, Libera Università Maria Santissima Assunta (LUMSA) University, Rome, Italy; ^2^Department of Psychology, Sapienza University, Rome, Italy; ^3^Departamento de Psicología Social, del Trabajo y Diferencial, Universidad Complutense, Madrid, Spain

**Keywords:** teaching, work engagement, quiet ego, ethical leadership, compassion at work

## Abstract

Recent revisions of the Job Demands Resources (JDR) model acknowledged the importance of personal and organizational dimensions enriching job resources’ effect on work engagement. Consistently, this paper addresses the role of compassion satisfaction, as a job resource, on teacher work engagement, given the saliency of caring in teaching as a helping profession. Furthermore, quiet ego, as a personal dimension, and ethical leadership, as an organizational dimension, are studied as antecedents of compassion satisfaction. Overall, the study verifies with a Structural Equation Model whether and how compassion satisfaction mediates the relationships among work engagement, quiet ego, and ethical leadership. One hundred and eighty-eight Italian teachers took part in the study by completing four scales: the Ethical Leadership Scale, the Quiet Ego scale, the Professional Quality Of Life Questionnaire, and the Utrecht Work Engagement Scale—ultra-short version. The final model showed a good fit to the data: χ^2^_(__48__)_ = 75.399, *p* = 0.007, CFI = 0.979, TLI = 0.971, RMSEA = 0.055 (90% CI = 0.029–0.078, *p* = 0.342), SRMR = 0.039. Findings showed that teachers’ compassion satisfaction is strongly related to their engagement at school, confirming that teachers’ care toward their students is an important resource supporting their engagement. Furthermore, compassion satisfaction totally mediates the relationship between quiet ego and work engagement (b_DIRECT_ = ns, b_INDIRECT_ = 0.327, *p* = 0.000). Such mediating path confirms recent expansions of the JDR model about the role of personal resources on job resources and, consequently, on work engagement and confirms the Conservation of Resources theory, stating that personal resources impact work outcomes. At the same time, compassion satisfaction does not mediate the relationship between ethical leadership and work engagement, so that ethical school leaders directly impact teachers’ work engagement. A possible reason for this finding relies on ethical leadership’s role in promoting higher school life participation as a community. More theoretical and practical implications are described in the paper.

## Introduction

Work engagement is a positive state of mind, characterized by vigor, dedication, and absorption, associated with optimal functioning at work (Schaufeli et al., 2002; Hakanen and Schaufeli, 2012). Considered as an indicator of well-being at work (Schaufeli et al., 2008), work engagement is studied in several occupational contexts, such as profit (Saks and Gruman, 2014; Gilsdorf et al., 2017; Sarwar et al., 2020), non-profit (Vecina et al., 2012, 2013; Dal Corso et al., 2019), health and social services (Mauno et al., 2016; Mostafa and Abed El-Motalib, 2020), educational institutions (Gozukara and Simsek, 2015; Fiorilli et al., 2019b, 2020). When addressing the educational field, the study of work engagement in teachers assumes a particular saliency, in consideration of the intrinsic motivation that characterizes the choice to become an educator (Darling-Hammond and Youngs, 2002; Han and Yin, 2016).

Studies on teachers’ work engagement considered several work-related resources when addressing its antecedents (Skaalvik and Skaalvik, 2014; Eldor and Shoshani, 2016; Minghui et al., 2018; Fiorilli et al., 2019b, 2020; Granziera and Perera, 2019; De Stasio et al., 2020). Such resources, for example, included social support at work (Minghui et al., 2018; Fiorilli et al., 2019b), person-job fit (Rothmann and Hamukang’andu, 2013; Li et al., 2015), or career perspectives (Timms and Brough, 2013). These works built on the Job Demands-Resources (JDR) theory, according to which job resources spark a motivational process in employees, so that the higher the job resources, the higher their energy and motivation toward work (i.e., work engagement), and, likely, the higher their chances to be productive and committed (Schaufeli, 2017). In this framework, indeed, job resources represent aspects of one’s job that may support the achievement of work goals, reduce job demands and the associated strain, or foster employee growth (Schaufeli, 2017). At the same time, the JDR theory individuates a risk path due to the job demands leading to burnout (Bakker and Demerouti, 2007; Schaufeli, 2017). Traditionally, studies on teachers’ well-being are focused on the latter path, namely the job demands-burnout relationship, individuating potential risk factors for teachers’ psychological health (Schwarzer and Hallum, 2008; Skaalvik and Skaalvik, 2009; Kinman et al., 2011; Pas et al., 2012; Buonomo et al., 2017), or protective factors against adverse consequences (Betoret, 2006; Skaalvik and Skaalvik, 2014; Van Maele and Van Houtte, 2015; Buonomo et al., 2017).

This work aims at contributing to the studies focusing on the motivational process described by the JDR, thus individuating factors enhancing teachers’ engagement (Li et al., 2015; Minghui et al., 2018; Zahed-Babelan et al., 2019; De Stasio et al., 2020).

More specifically, our paper indicates compassion satisfaction as a possible job resource impacting teachers’ work engagement. Compassion satisfaction refers to the sense of accomplishment and reward related to the compassionate care given to others in one’s job, as connected with acknowledging its effectiveness (Stamm, 2009). A worker is defined as “compassionate” when he/she perceives others’ suffering, acknowledges its causes, and is motivated to take action and help reduce it (Atkins and Parker, 2012). Compassion satisfaction is frequently studied in medical helping professionals (Mason et al., 2014; Meyer et al., 2015; Kim et al., 2017; Vagharseyyedin et al., 2018; Zhang et al., 2018; Ortega-Campos et al., 2020). At the same time, an emerging interest to study compassion satisfaction in other professional categories arose lately, with specific regard to other types of helping professionals, such as police officers (Chiappo-West, 2017), residential child care professionals (Audin et al., 2018), and teachers (Christian-Brandt et al., 2020). Overall, such studies inform about the protective, resourceful impact of compassion satisfaction on job beliefs and behaviors.

More recent revisions of the JDR acknowledged that other aspects of daily life might influence employee perceived job resources (Schaufeli, 2017). Such dimensions include both personal dispositions, traits, and characteristics (Xanthopoulou et al., 2007, 2009; Schaufeli, 2017), as well as organizational features (e.g., leadership styles, organizational support; Albrecht and Andreetta, 2011; Tuckey et al., 2013; Schaufeli, 2015, 2017). In the current paper, two antecedents of job resources (i.e., compassion satisfaction) are considered: Quiet ego as a personal antecedent and ethical leadership as an organizational antecedent. Authors chose these constructs due to the acknowledgment that teachers are helping professionals and, accordingly, give added saliency to the relational aspects and implications of their job tasks (O’Connor, 2008; Pereira et al., 2015; Eldor and Shoshani, 2016).

Quiet ego is a self-identity in which concerns for the self and others are balanced, and both one’s own’s and others’ growth is promoted (Wayment et al., 2011, 2015; Wayment and Bauer, 2018). Bauer and Wayment described the quiet ego as “an identity that incorporates others without losing the self” (Bauer and Wayment, 2009). Quiet ego is defined by two main stances: (1) balance, intended as the ability to take into account needs, desires, and point of view of the self and others, and to consider their positive and negative aspects; (2) growth, intended as the ability to develop adaptively on the long term, instead of satisfying contextual desires (Wayment et al., 2015). In other words, people with high levels of quiet ego are prone to include others’ instances in their cognitive and emotional processes and care about them while not losing self-attunement and self-caring (Wayment et al., 2015). Previous studies showed that such individuals are more likely to score high in several well-being measures, such as self-esteem, savoring, life satisfaction, and subjective well-being (Wayment et al., 2011, 2015; Wayment and Bauer, 2018).

Overall, such theory suggests that, when individuals have a quiet ego, they are more prone to engage in caring about others, support them, and acknowledge their desires, while avoiding depletion of personal resources (Wayment and Bauer, 2017). Few studies considered such relationships at the workplace (Huffman et al., 2015; Wayment et al., 2019). At the same time, studying the relationship between personal dispositions toward others (i.e., quiet ego) at work is interesting, above all considering the protective role they are usually ascribed to when it comes to prevent adverse work conditions and promote well-being and engagement (e.g., Aw et al., 2020). In the context of this study, the “quiet” identity will be linked with satisfaction due to the rewards after caring about other organizational actors (i.e., compassion satisfaction). Such an approach would deepen our knowledge about other organizational actors’ relationship effects on workers’ well-being and attitudes toward their job experience. More specifically, considered the saliency of caring in the teaching profession (O’Connor, 2008), we want to explore the links between quiet ego and compassion satisfaction in a group of teachers.

Among constructs influencing job resources, organizational dimensions have been recognized a prominent role (Schaufeli, 2017). Workers, indeed, do not work in a vacuum. The deep link with the organizational context is specifically relevant for teachers. Several studies showed that school-related variables (e.g., leadership, collective efficacy, school climate) have a role in how teacher perceive and whether they are engaged in their job (Ware and Kitsantas, 2007; Toytok and Kapusuzoglu, 2016; Koutouzis and Malliara, 2017; Benevene et al., 2019; Fiorilli et al., 2019b; Buonomo et al., 2020b). Among these variables, school principals’ ethical leadership may sustain teachers’ proneness to acknowledge others’ needs and support them (Brown and Treviño, 2006). Ethical leadership, indeed, is “the demonstration of normatively appropriate conduct through personal actions and interpersonal relationships, and the promotion of such conduct to followers through two-way communication, reinforcement, and decision-making” (Brown et al., 2005, p. 120). Such definition encloses three main aspects (Brown et al., 2005; Brown and Treviño, 2006; Benevene et al., 2018). More specifically, ethical leaders should: (1) define an ethical standard; (2) behave accordingly, by making decisions and rewarding who follows such standards; (3) communicate on ethical issues raised by the work in the organization openly, by allowing the employees to express their points of view. According to Brown and Treviño, ethical leaders’ impact on employees’ values and behavioral standards may strengthen their prosocial attitudes at work, either because ethical leaders act as role models, or workers are motivated to reciprocate leaders behaviors (Brown and Treviño, 2006). This consideration is consistent with the Conservation of Resources (COR) theory (Hobfoll, 1989, 2002; Hobfoll and Shirom, 2001), claiming that every individual is motivated to gain and retain resources, such as energies, objects, or skills (Hobfoll, 2002). According to the COR theory, employees with higher resources are not only more prone to deal with unexpected demands and strain but even more likely to gain other resources, creating a gain spiral (Llorens et al., 2007; Salanova et al., 2010). In other words, the COR theory showed that resources do not exist in isolation but are aggregated (Hobfoll, 2002). According to Salanova and colleagues (Salanova et al., 2013), in terms of job well-being, this means that when employees work in a resourceful workplace, they are more likely to be good workers because their positive beliefs and emotions toward work (e.g., work engagement) are promoted and sustained (Salanova et al., 2010). This point suggests that effective, positive leadership could broaden employees’ resources at work, such as compassion satisfaction and work engagement. Furthermore, the COR model posits that personal life and work-life resources are reciprocal and foster one another (Hobfoll, 2002; Salanova et al., 2010). Consistently, positive personal resources (i.e., quiet ego) may enhance resources at work.

Overall, this work contributes to apply JDR and COR theory to the teachers’ work experience, considering whether and how compassion satisfaction (as a job resource) mediated the relationship of quiet ego (as a personal antecedent) and ethical leadership (as a work-related antecedent) on work engagement.

### Compassion Satisfaction and Work Engagement

Studies addressing the consequences of compassion satisfaction on employees’ life show a positive association between this construct and positive attitudes toward work, included work engagement (Mason et al., 2014; Meyer et al., 2015; Chiappo-West, 2017; Kim et al., 2017; Audin et al., 2018). Among the mentioned studies, the only one involving teachers did not explore the associations with work engagement and was explicitly related to the care of children with trauma histories (Christian-Brandt et al., 2020). At the same time, studies on the role of caring in teaching inform about the strong motivation in schoolteachers to aim for children’s growth, support, and development (Sutton and Wheatley, 2003; O’Connor, 2008; Eldor and Shoshani, 2016; Buonomo et al., 2019). Such studies suggest that teachers are likely to experience compassion satisfaction and benefit from it.

To the best of our knowledge, studies taking into account work engagement in other professions did not address the associations between compassion satisfaction and each dimension of work engagement (i.e., vigor, dedication, absorption). Nonetheless, it is possible to individuate some mechanisms explaining why compassion satisfaction and work engagement are related. With this regard, some authors claimed that positive emotions are a common point between compassion satisfaction and work engagement (Fredrickson and Losada, 2005; Bakker and Demerouti, 2008; Audin et al., 2018). According to the Broaden and build theory, indeed, workers experiencing positive emotions can broaden their behavioral and cognitive repertoire, and, as a result, work better and more intensely (Fredrickson, 2001). In other words, according to this theory, the positive emotions related to compassion satisfaction may enhance the workers’ amount of vigor and absorption, respectively, described as “high levels of energy and mental resilience while working” and “being fully concentrated and happily engrossed in one’s work” (Bakker and Demerouti, 2008, p. 2). At the same time, Fredrickson’s theory acknowledges the “upward spiral” effect of positive emotions, namely the ability of positive experiences, if repeated over time, to promote emotional well-being, thus resulting in a higher ability to cope with difficulties and give meaning to their experience. In other words, according to the upward spiral effect, the positive emotions related to compassion satisfaction may enhance the workers’ amount of dedication, described as “being strongly involved in one’s work and experiencing a sense of significance, enthusiasm, and challenge” (Bakker and Demerouti, 2008, p. 2).

Overall, previous studies inform about the potential connections between compassion satisfaction and work engagement in teachers. Thus, the following hypothesis was formulated:

H1. Compassion satisfaction is positively related to teachers’ work engagement.

### Quiet Ego, Compassion Satisfaction, and Work Engagement

Although most studies on the quiet ego were not specifically focused on work-life, some recent contributions shed new light on the quiet ego’s role in organizations. More specifically, Huffman et al. (2015) and Wayment et al. (2019) underlined that applying the quiet ego construct to organizational life may help practitioners and researchers to vehiculate the importance of balancing the concerns for the self and others for healthier work-life. Furthermore, the authors claimed that quiet ego-based interventions strengthen the relationships between employees and organizations by fostering workers’ positive attitudes toward the organization and compassion toward their colleagues, and, ultimately, organizational knowledge and performance (Huffman et al., 2015). This hypothesis was confirmed in a brief quiet ego intervention held in a group of nurses, where quiet ego-related abilities were associated with lower fatigue due to the care work (Wayment et al., 2019). Overall, the few studies considering the role of quiet ego in organizational contexts suggest that employees with high quiet ego levels feel better at work. These first studies may follow the research direction according to which personal resources impact job resources related to work engagement, as claimed in the JDR model revision (Schaufeli, 2017), and as suggested by the COR theory (Salanova et al., 2010). At the same time, as described, studies on the general population show a link between quiet ego and well-being measures. To the best of our knowledge, current research did not focus on the implication of quiet ego research (or interventions) on schoolteachers, but, building on the mentioned studies, we could hypothesize that:

H2. Quiet ego has a positive association with teachers’ work engagement

At the same time, several studies showed that the association between quiet ego and compassionate care is embedded in the definition of the quiet ego itself (Bauer and Wayment, 2009; Wayment et al., 2015; Wayment and Bauer, 2018). Wayment, indeed, defines the quiet ego as a compassionate self-identity (Wayment et al., 2015). Consistently, it seems reasonable that being highly attuned to compassionate care (because of quiet ego) could be linked to good satisfaction levels for one’s caring behaviors. This idea is consistent with what Bauer and Wayment claimed in 2017: “When a person’s ego is quiet, that person is motivated and able […] to view a situation as an opportunity for prosocial development” (Wayment and Bauer, 2017). Therefore, our third hypothesis is:

H3. Quiet ego is positively associated with teachers’ compassion satisfaction.

### Ethical Leadership, Compassion Satisfaction, and Work Engagement

Several studies claimed that the peculiar behaviors (i.e., explicit ethical and value-related messages, intentional role modeling of ethical behaviors, use of reward system to reinforce ethical behaviors) shown by ethical leaders foster employee engagement, either because they share a clear behavioral standard or because they proactively involve workers in the organizational decision making (Engelbrecht et al., 2014; Asif et al., 2019; Mostafa and Abed El-Motalib, 2020; Sarwar et al., 2020; Zeng and Xu, 2020). Overall, such studies confirm the recent extension of the JDR model, according to which leadership may be considered an antecedent of job resources impacting work engagement (Schaufeli, 2017), and the COR theory, as it confirms the creation of gain spirals for employees inserted in a positive workplace (Salanova et al., 2010). The relationship between ethical leadership and work engagement was studied in several organizations, including public (Asif et al., 2019; Mostafa and Abed El-Motalib, 2020) and private (Sarwar et al., 2020) sectors, as well as university teachers (Zeng and Xu, 2020) and helping professionals (Mostafa and Abed El-Motalib, 2020). To the best of our knowledge, previous studies did not consider the relationship between ethical leadership and work engagement in schoolteachers.

Furthermore, most previous studies addressed the links among ethical leadership, work engagement, and organization-related outcomes or antecedents, such as organizational trust, organizational performance, or affective commitment (Asif et al., 2019; Sarwar et al., 2020; Zeng and Xu, 2020), lacking to verify its effect on personal or relational resources at work. At the same time, building on studies on teachers in higher education and helping professionals, it could be hypothesized that ethical leadership boosts teachers’ work engagement. Thus, our hypothesis is that:

H4. Principals’ ethical leadership has a positive association with teachers’ work engagement

Furthermore, literature on ethical leadership shows an association with compassion satisfaction. A seminal work by Brown and Treviño (2006) suggested that ethical leadership is connected with prosocial behaviors at work. The authors provide two possible mechanisms underlining such link: Social learning processes, in which ethical leaders acts as legitimate role models; and social exchange processes, in which worker behave prosocially in light of the trusting, caring and fair treatment they receive from the leader. Consistently, in a recent paper, Zoghbi-Manrique-de-Lara and Viera-Armas (2019) showed that having an ethical leader at work fosters organizational citizenship behaviors toward colleagues in the form of delivering support for their well-being. While these studies suggest a relationship between ethical leadership and supportive behaviors at work, there are no research contributions, to the best of our knowledge, on the relationship between ethical leadership and compassion toward users, patients, clients, or, in the case of teachers, students. At the same time, in consideration of the centrality of caring in the teaching profession, we could expect that:

H5. School principals’ ethical leadership (as perceived by the teachers) is positively associated with teachers’ compassion satisfaction.

Overall, the potentially crucial role of compassion satisfaction, even supported by several studies on the centrality of caring in the teaching profession, suggests that this construct may mediate the impact of both organizational (i.e., ethical leadership) and personal (i.e., quiet ego) resources on work engagement. Thus, the final hypothesis is:

H6. Compassion satisfaction mediated the relationship of school principals’ ethical leadership (as perceived by the teachers) and quiet ego with work engagement.

**FIGURE 1:**
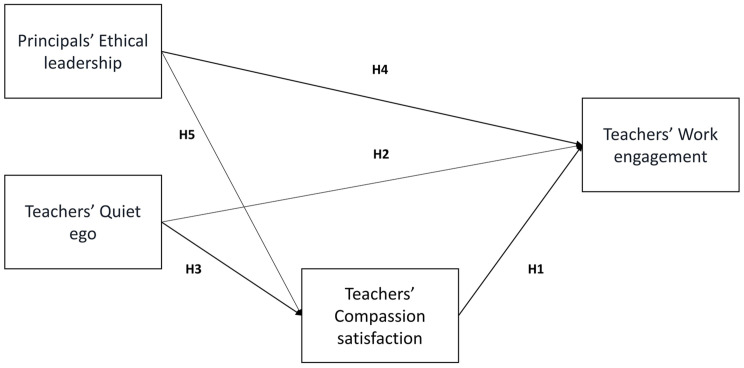
Theoretical model and hypotheses. H6 (Compassion satisfaction mediated the relationship of ethical leadership and quiet ego with work engagement) corresponds to the combination of H1, H3, and H5.

Figure 1 synthesizes the hypotheses.

## Materials and Methods

### Participants and Procedure

One hundred and eighty-eight teachers (Female = 85%) from primary and secondary schools in Italy were recruited for this study. They were aged 36–61 years (*M* = 49.72, *SD* = 8.87), and more than 70% had a university degree or equivalent. Regarding their work contexts, participants taught in their current schools for 0–36 years (*M* = 11.73, *SD* = 9.46), and more than 80% had a permanent contract. Finally, 60% of the teachers taught in a primary school.

Data were gathered online through survey software. The administration of the protocol was anonymous and confidential. Participants were asked to sign an informed consent, which clarified that the research group was independent of their organization and that only the researchers would have had access to the data. Overall, these procedures would address a potential social desirability bias.

### Measures

This study involves four variables: Ethical leadership, quiet ego, compassion satisfaction, and work engagement.

Ethical leadership was assessed with the Ethical Leadership Scale (Brown and Treviño, 2006), which includes ten items, measured on a 5-point Likert scale (1 = Totally disagree, 5 = Totally agree). A sample item is: “Our school principal makes fair and balanced decisions” (item 5). In this study, Cronbach’s alpha was 0.961, slightly higher than the internal consistency reported by Brown and Treviño(2006; alpha = 0.92).

Quiet ego was measured with the Quiet Ego Scale (Wayment et al., 2015), which includes 14 items, measured on a 5-point Likert scale (1 = Totally disagree, 5 = Totally agree). A sample item is: “Before criticizing somebody, I try to imagine how I would feel if I were in their place” (item 4). In this study, Cronbach’s alpha was 0.720, slightly lower than the internal consistency reported by Wayment and colleagues (2015; alpha = 0.78).

Compassion satisfaction was measured with a subscale from the Professional Quality Of Life Questionnaire (ProQOL) (Stamm, 2009), which includes 10 items, measured on a 5-point Likert scale (1 = Totally disagree, 5 = Totally agree). This tool is commonly used when evaluating the experience of professionals, like teachers, who provide care to others as part of their job (Pereira et al., 2015). A sample item is: “I get satisfaction from being able to help students” (item 1). Each time the original version of the questionnaire referred to people or patients, the participants were asked about their students instead. In this study, Cronbach’s alpha was 0.888, thus confirming the internal consistency reported by Stamm(2010; alpha = 0.88).

Work engagement was assessed with the ultra-short version of the UWES scale (Schaufeli et al., 2019), which includes three items (one for each work engagement subscale), measured on a 5-point Likert scale (1 = Totally disagree, 5 = Totally agree). A sample item is: “I am enthusiastic about my job” (item 2). In this study, Cronbach’s alpha was 0.844, thus confirming the range of internal consistency measures reported by Schaufeli and colleagues (2019; alpha ranging from 0.77 to 0.88).

Finally, sociodemographic (age, gender, educational level) and work-related (years of experience, type of contract, type of school) data were gathered with a brief *ad hoc* questionnaire.

### Data Analysis

Firstly, three procedures of data exploration were implemented: (a) univariate and multivariate outlier detection, with Mahalanobis’s distance set to *p* < 0.001 (Gath and Hayes, 2006); (b) score distribution analysis, with skewness and kurtosis cut-off points set to [-2; + 2] (George and Mallery, 2003); (c) missing value analyses (when present, they were omitted listwise) (Little, 1992). After these procedures we deleted 23 subjects, and obtained the sample described in section “Participants and Procedure.”

Secondly, to test the common method variance bias, the Harman’s single-factor test was pursued. Findings showed that the single factor emerging from the exploratory factor analysis only accounted for the 22% of the covariance among the measures, meaning that the are no issues associated with common method variance in the data (Podsakoff and Organ, 1986).

Pearson’s correlations were measured among work engagement, ethical leadership, quiet ego, and compassion satisfaction to verify the associations between the variables and such constructs and demographics (age, gender) and work-related variables (years of experience, primary vs. secondary school).

After such preliminary analyses, a Confirmatory Factor Analysis (CFA) (Kline, 2011) was performed in order to examine the measurement model with MPlus version 8 (Muthén and Muthén, 2017). Three item parcels were created for all the administered measures, except for the UWES ultra-short scale, to enhance our model’s reliability and parsimony. Each parcel was created by sequentially summing items assigned based on the highest to lowest item-total corrected correlations (Little et al., 2002, 2013; Coffman and MacCallum, 2005). Parceling allows obtaining less free parameters to estimate and reduce sampling error sources (Little et al., 2002, 2013; Coffman and MacCallum, 2005). The Robust Maximum Likelihood Approach (MLR) was used to deal with non-normality in data (Wang and Wang, 2012).

Next, the structural equation modeling (SEM) approach (Kline, 2011) was implemented. Under the model, Ethical leadership and Quiet ego were directly and indirectly (through Compassion satisfaction) associated with Work engagement. According to a multifaceted approach to the assessment of the fit of the model (Tanaka, 1993), the following indices were used to evaluate the goodness-of-fit: The Chi-square likelihood ratio statistic, the Tucker and Lewis Index (TLI), the Comparative Fit Index (CFI), the Root Mean Square Error of Approximation (RMSEA), with its confidence intervals, and the Standardized Root Mean Square Residual (SRMR). It has to be noted that, according to Kline (2015), the Chi-square index could result significant because of its sample size-sensitive bias. We accepted TLI and CFI values greater than 0.95 (Hu and Bentler, 1998), RMSEA values lower than 0.08 (Browne and Cudeck, 1992; Hooper et al., 2008), and SRMR values lower than 0.08 (Hu and Bentler, 1998; Hooper et al., 2008).

## Results

### Measurement Model and Correlations Among Work Engagement, Ethical Leadership, Quiet Ego, and Compassion Satisfaction

The measurement model showed a good fit to the data: χ^2^(48) = 75.399, *p* = 0.007, *CFI* = 0.979, *TLI* = 0.971, *RMSEA* = 0.055 (90% CI = 0.029–0.078, *p* = 0.342), *SRMR* = 0.039. Table 1 shows the correlations among Ethical leadership, Quiet ego, Compassion satisfaction, and Work engagement. As expected, Ethical leadership showed a positive correlation with both Compassion satisfaction (*r* = 0.218, *p* < 0.05) and Work engagement (*r* = 0.256, *p* < 0.01). Quiet ego showed positive significant associations with Compassion satisfaction (*r* = 0.476, *p* < 0.01) and Work engagement (*r* = 0.424, *p* < 0.01), too. Correlations among such variables and demographic and work-related variables are not shown, as they were not significant (*p* > 0.05).

**TABLE 1:** Correlations among work engagement, ethical leadership, quiet ego, and compassion satisfaction.

Variables	1.	2.	3.	4.
1. Work engagement	–	0.256**	0.424**	0.718**
Ethical leadership		–	0.151	0.218*
Quiet ego			–	0.476**
Compassion satisfaction				–

**p < 0.01, *p < 0.05.

### Final Model

**FIGURE 2:**
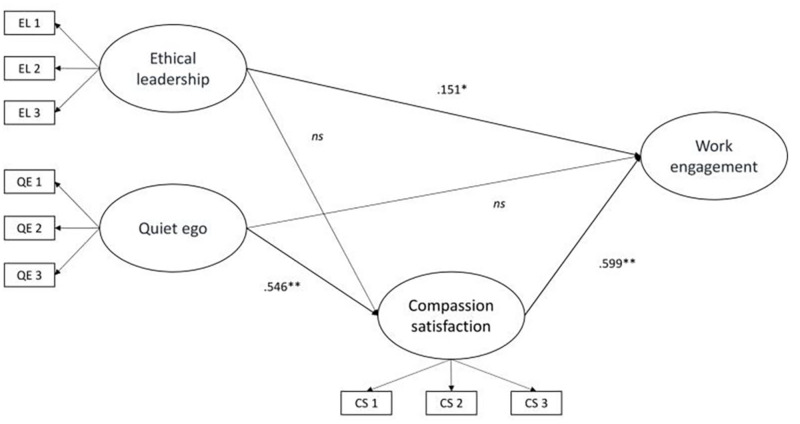
Results of the structural equation model. Standardized direct effects were reported. ^∗^*p* < 0.05, ^∗∗^*p* < 0.01. EL, Ethical Leadership; QE, Quiet Ego; CS, Compassion Satisfaction.

The final model (shown in Figure 2), showed a good fit to the data: χ^2^(48) = 75.399, *p* = 0.007, *CFI* = 0.979, *TLI* = 0.971, *RMSEA* = 0.055 (90% CI = 0.029–0.078, *p* = 0.342), *SRMR* = 0.039. The model showed that Quiet ego is positively associated with Compassion satisfaction (*b* = 0.546, *p* < 0.001), and Compassion satisfaction with Work engagement (*b* = 0.599, *p* < 0.001), thus confirming H3 and H1.

The direct association between quiet ego and work engagement (H2) was not confirmed, as these relationships were not significant in the final model. Conversely, findings confirmed an indirect relation, showing that Compassion satisfaction fully mediated the relationship between Quiet ego and Work engagement (*b*_*DIRECT*_ = ns, *b*_*INDIRECT*_ = 0.327, *p* = 0.000).

The model also showed that Ethical leadership is positively associated with Work engagement (*b* = 0.151, *p* < 0.05), thus confirming H4. The direct association between Ethical leadership and compassion (H5) was not confirmed, as this relationship was not significant in the final model.

Furthermore, findings showed that Compassion satisfaction did not mediate the relationship between Ethical leadership and Work engagement (*b*_*INDIRECT*_ = ns). Thus, overall, H6 is partially confirmed.

The percentages of variance explained were 32.8% for Compassion satisfaction and 44.3% for Work engagement.

## Discussion

Results confirmed most of our hypotheses. More specifically, findings showed that teachers’ compassion satisfaction is strongly related to high engagement at school (H1), thus suggesting that teachers’ care toward their students is a crucial resource supporting their engagement. Furthermore, the findings showed that the observed association between quiet ego and work engagement (H2) is totally mediated by compassion satisfaction (partially confirming H6), so that quiet ego contributes to a higher compassion satisfaction (H3), and this, in turn, is connected to higher engagement. Finally, compassion satisfaction does not mediate the relationship between ethical leadership and work engagement (partially disconfirming H6 and H5), so that having ethical school leaders directly impacts teachers’ work engagement (H4). The following sections will describe the theoretical and practical implications of such results.

Regarding the mediating role of compassion satisfaction, the first result to be addressed is the total mediation of compassion satisfaction in quiet ego effect on work engagement. Building on the acknowledgment that being compassionate requires not only to feel others’ suffering but also to feel the need to reduce their pain, this result suggests that compassion satisfaction may sustain teacher’s motivation to care about their students, and this, in turn, may strengthen their engagement at school. While the quiet ego foresees a “compassionate” dimension (Wayment et al., 2015), it is interesting to note that even compassion satisfaction has a strong relationship with it. From our results, it seems that being motivated toward other people’s growth and needs (i.e., being compassionate) and even satisfied about one’s own effectiveness in caring about others are deeply connected to the quiet ego. Overall, such a finding confirms Wayment and Bauer (2017) idea that quiet ego motivates people to perceive interactions as prosocial development opportunities. As professionals involved in students’ growth and development (O’Connor, 2008; Eldor and Shoshani, 2016), it is possible that teachers find in compassion satisfaction at school a way to express and commit to their growth and engagement, too. Reframing compassion satisfaction in terms of balance between self and others (as for the quiet ego construct), the personal, self-related sense of accomplishment and pleasure expressed by this construct is balanced by the source of such well-being, namely others and the chance to care about and support them (Stamm, 2009). Overall, our findings suggest that compassion satisfaction may be a work-related experience strongly connected with the dispositional, personal quiet ego construct. Further research could generalize this association, defining, for example, whether and how other helping professionals may show similar findings. At the same time, this mediating path confirms the JDR model’s recent expansions about personal resources’ role on job resources and, consequently, on work engagement (Schaufeli, 2017). Previous studies, indeed, included personality characteristics among such personal resources, using them in the model as antecedents of job resources (Xanthopoulou et al., 2007; Schaufeli, 2017). Consistently, the quiet ego was described as a characteristic adaptation, namely a domain of personality that focuses on values and motives (McAdams, 1995), which, in the case of quiet ego, are related to self and others’ balance and growth (Wayment and Bauer, 2017). Furthermore, this result endorses Salanova et al. (2010) claim on the COR theory regarding personal resources’ impact in work contexts. From a practical point of view, these findings connect with the attention for compassion-based interventions in healthcare organizations. Such interventions may address compassion at different levels of the workplace experience: Individual (on self-compassion and compassion toward others; Scarlet et al., 2017; Wayment et al., 2019), leadership (on the construction of a compassionate leadership style; de Zulueta, 2016), teams (on reflective and supporting compassionate activities, Pepper et al., 2012; Barker et al., 2016), whole organizations (on compassionate culture and values; de Zulueta, 2016). As extensively reported in the literature, building compassionate workplaces boosts employees’ well-being and organizational performance (West et al., 2015; de Zulueta, 2016; Wayment et al., 2019). Further studies could extend such protocols in the educational field. Several studies, indeed, show that social support, empathy and caring among colleagues and from principals at school improves teacher well-being (Pas et al., 2012; Ross et al., 2012; Fiorilli et al.,2019a,b) and productivity (Chan, 2004; Cosner, 2009).

Findings showed a higher significance of the path involving personal resources (i.e., quiet ego) than organizational resources (i.e., ethical leadership). Ethical leadership, indeed, shows poor correlations with compassion satisfaction. Consistently, the final model lacks a mediating effect of compassion satisfaction in the relationship between ethical leadership and work engagement. This finding can be read in the light of current knowledge regarding the prosocial consequences of having an ethical leader. According to the literature addressing this topic, the equity-, group-oriented approach to leadership proposed by ethical leaders may enhance employees’ ability to acknowledge others’ points of view when making important decisions and confronting ethical issues, but not necessarily when considering the caring dimension of work (Brown and Treviño, 2006; Zoghbi-Manrique-de-Lara and Viera-Armas, 2019). Further study could explore whether and how other leadership styles may be more effective for this kind of process. For example, this could be the case for compassionate leadership (West et al., 2015; de Zulueta, 2016), focused on a self-disclosing, caring attitude toward others. Likely, the lack of a clear focus on caring and support in work relationships may explain the missed link between ethical leadership and the feeling of accomplishment from caring for the students. At the same time, the construct of compassion satisfaction regards the satisfying effects of being compassionate toward students, not the compassionate acts themselves. Further studies may consider such a relationship and explore potential mediators of the ethical leadership—compassion (or compassion satisfaction) link.

Apart from the effects of compassion satisfaction, our results underline the role of ethical leadership in enhancing teachers’ work engagement, confirming our hypothesis. In this study, ethical leadership has a unique, although weak, impact on work engagement, even when considering the satisfaction of the helping relationship toward students. This consideration raises a question about the role of organizational dimensions on teachers’ work and occupational well-being. Despite some studies considered such links before (Ware and Kitsantas, 2007; Klassen et al., 2010; Buonomo et al.,2020a,b), most studies on teachers’ well-being and motivation at school focus either on personal variables or on the teacher-student relationship. This tendency is partially due to the teacher autonomy in choosing teaching methods and strategies within the class, which is even legally regulated by the Italian Law (Legislative Decree 297/1994). Such autonomy likely influences teachers’ perceptions of the leader role so that he/she is deemed more as a coordinator than a manager. At the same time, teaching does not occur in isolation because of the presence of a teaching community lead by a principal, as well as of the intricate web of bureaucracy and governmental requirements that regulate the organizational structure and processes within the school (OECD, 2014; Ainley and Carstens, 2018). Thus, a leader managing such collective dimensions by providing and embodying ethical behavioral standards, promoting an open discussion about concerns and needs, and sharing his/her decision-making processes with the school staff, may help teachers feel more attuned to the collective aspects of their job. In other words, ethical leaders may impact school climate and values (e.g., Enwereuzor et al., 2020). In turn, the higher contribution to the school life as a community (integrated to the teacher-student or teacher-class point of view) could enhance teacher work engagement. Consistently, the few studies addressing the role of collective dimensions on teachers’ work engagement show the unique impact of dimensions like school culture (Zahed-Babelan et al., 2019) and goal structures (Ciani et al., 2008; Skaalvik and Skaalvik, 2013) on teachers’ motivation and engagement toward their job. Further studies could verify, first, the role of ethical leadership in promoting teachers’ work engagement and compassion satisfaction with a multi-level approach. This would allow researchers to verify whether the peculiar organizational context would impact such relationships. Secondly, it would be valuable to focus on the theoretical implications and the practical consequences of delivering an ethical leadership-based intervention at school. Such intervention could address deans and their staff and take into account moral reasoning, decision-making processes, interpersonal skills, and, finally, job-related identities (Brown and Treviño, 2006).

Despite the suggestions and implications related to our findings, this work is not without limitations. Firstly, our results are driven on nation-based, cross-sectional data from a relatively small sample. From one side, longitudinal and qualitative works could provide a more deepened understanding of the relationships among the constructs. From the other, it is crucial to acknowledge that a bigger sample would have allowed for a higher representativeness, as well as for comparisons among, for example, teachers working in different types of schools, with different years of experience, or different type of contracts. Furthermore, as all the participants are from Italy, it is likely that the results are influenced by macrosocial and cultural dynamics, too. Secondly, the lack of a multi-informant source for our data may lead to common method bias. At the same time, we tested the significance of this issue with the Harman’s single-factor test, and applied some suggestions from Conway and Lance (e.g., preservation of anonymity, removal of unengaged/outlier responses, testing each scale reliability and the general measurement model) (Conway and Lance, 2010) to address this issue. Nevertheless, it would be interesting to address the role of principals’ leadership styles as perceived by teachers, but even as described by principals themselves. Thirdly, a comparison among the effects of ethical leadership and other leadership styles, such as compassionate, empowering, or transformational leadership, would have helped to clarify the associations between teachers’ perceptions about principals’ leadership styles and their engagement levels, giving a higher contribution to the role of leadership as an antecedent of job resources in the JDR model. Considering the care-centered approach to work frequently reported in teaching, it is likely that other leadership styles would have had a different impact on the studied variables. Finally, the spiral effects postulated in the COR theory, which could be the basis of the leadership-compassion satisfaction-work engagement path reported in the study, could be further analyzed with the implementation of multi-level studies. As per the COR theory recognizes the importance of aggregated personal and organizational resources at work in predicting job well-being, multi-level models would help clarifying how resourceful workplaces explain individuals’ well-being at work, while taking into account personal resources as well (Hardy and Bryman, 2012).

## Data Availability Statement

The datasets generated for this study are available on request to the corresponding author.

## Ethics Statement

The studies involving human participants were reviewed and approved by the Ethical Committee for Scientific Research at LUMSA University. The patients/participants provided their written informed consent to participate in this study.

## Author Contributions

IB and PB: study conceptualization and construction. IB and MLV: methodology. IB: statistical analyses. IB and MLF: writing—original draft preparation. IB, PB, MLF, and MLV: writing—review and editing. All authors have read and agreed to the published version of the manuscript.

## Conflict of Interest

The authors declare that the research was conducted in the absence of any commercial or financial relationships that could be construed as a potential conflict of interest.
